# Comparative Metabolic Profiling in *Drosophila suzukii* by Combined Treatment of Fumigant Phosphine and Low Temperature

**DOI:** 10.3390/metabo14100526

**Published:** 2024-09-28

**Authors:** Junbeom Lee, Hyun-Kyung Kim, Jong-Chan Jeon, Seung-Ju Seok, Gil-Hah Kim, Hyun-Na Koo, Dae-Weon Lee

**Affiliations:** 1 Metabolomics Research Center for Functional Materials, Kyungsung University, Busan 48434, Republic of Korea; 2 Department of Plant Medicine, College of Agriculture, Life and Environment Science, Chungbuk National University, Cheongju 28644, Republic of Korea; 3 Department of SmartBio, Kyungsung University, Busan 48434, Republic of Korea

**Keywords:** *Drosophila suzukii*, phosphine, low temperature, metabolomics, lipidomics

## Abstract

**Background/Objectives:** The mechanisms of action of phosphine are diverse and include neurotoxicity, metabolic inhibition, and oxidative stress; however, its efficacy at low temperatures is unclear. **Methods**: Comparative metabolomics is suitable for investigating the response of the spotted-wing fly *Drosophila suzukii* to exposure toward a combination of cold stimuli and fumigant PH_3_. **Results**: Under this combined exposure, 52 metabolites exhibiting significant differences in stress were identified and their physiological roles were analyzed in the *Drosophila* metabolic pathway. Most metabolites were involved in amino acids, TCA cycle, and nucleic acids. In addition, the alteration levels of cell membrane lipids, such as glycerophospholipids, sphingolipids, and glycerolipids, clearly showed changes in the combined treatment compared to PH_3_ and low temperatures alone. Aconitic acid, a component of the TCA cycle, was completely inhibited by the combined treatment. **Conclusions**: These results suggest that treatment-specific indicators could be useful biomarkers to indicate the synergistic effects of PH_3_ and low temperature on energy metabolism.

## 1. Introduction

The spotted-wing fly *Drosophila suzukii* (Matsumara) is widely distributed in Asia, the Americas, and Europe, and is characterized by laying eggs inside fresh fruits using serrated ovipositors [1,2,3,4,5]. Since hatched larvae burrow into the fruit, they are difficult to detect during the early stages of infection [6,7,8,9]. Wounds caused by female ovipositing organs are entry points for secondary pathogens, such as fungi and bacteria, which further aggravate fruit damage [6,7,8]. This invasive behavior has made this species a serious pest worldwide, and its control is crucial at the quarantine stage [10,11,12,13].

Phosphine (PH_3_) as a fumigant has been widely used to control pests in stored grains and many other stored commodities [14], but its mode of action is not well understood. PH_3_, which is broken down into harmless phosphates, is very effective for controlling pests in grain storage when used as a combined treatment with carbon dioxide [15,16,17]. Although effective penetration into target pests, lack of residue, and low cost are the major advantages of PH_3_ [18,19,20], long-term exposure is considered a weak point [21].

Low temperatures are primarily used to disinfect stored agricultural products from pests or for quarantine purposes [22,23,24]. Cold treatment has been used as a control measure against pests such as the Mediterranean fruit fly *Ceratitis capitata* (Wiedemann) (Diptera: Tephritidae) [25] and Caribbean fruit fly *Anastrepha suspensa* (Loew) (Diptera: Tephritidae) [26]. In addition, cold treatment significantly reduced adult emergence in both blueberries and strawberries, and extended the shelf life of infested fruits compared to untreated controls [27]. Therefore, low temperatures can be a good option not only for pest control, but also for maintaining the marketability of fruits [27,28].

Recent studies on fumigants have suggested the possibility of controlling pests by using combined treatments at low temperatures. Cold treatment increases the insecticidal activity of phosphine and ethyl formate against pests such as *D. suzukii* [2,5,29], the oriental fruit fly *Bactrocera dorsalis* (Hendel) (Diptera: Tephritidae) [30], and the peach fruit moth *Carposina niponensis* (Lepidoptera: Carposinadae) [31]. As cold treatment is commonly performed to maintain the marketability of fruits and vegetables, combined treatment with fumigants has the advantages of time efficiency, reduced product damage, and easy control of processing conditions. Although cold fumigation has a synergistic effect on insecticidal activity, the factors that lead to this synergistic effect are not well understood. To obtain evidence of the synergistic effects in pest management, the molecular changes induced by cold conditions and PH_3_ were investigated using comparative metabolic profiling.

## 2. Materials and Methods

### 2.1. Insect Rearing

The spotted-wing fly, *D. suzukii* (Matsumura) (Diptera: Drosophilidae), was provided by Dr. Bong-Su Kim (Plant Quarantine Technology Center, Animal and Plant Quarantine Agency, Gimcheon, Republic of Korea). *D. suzukii* was reared in the insect chamber at 20 ± 1 °C and 60 ± 10% relative humidity under a photo-period of 16 h light and 8 h dark [2,5,32]. The insects were maintained in a clean breeding dish (ø 100 mm × h 40 mm) supplied with artificial food and distilled water containing 20% sugar.

### 2.2. Phosphine and Thermal Treatment

PH_3_ (Vivakill^®^, 2% PH_3_ + 98% CO_2_) was purchased from Dongbu Farm Hannong Co., Ltd. (Daejeon, Republic of Korea) and supplied by Safefume Co., Ltd. (Fumate™, 99%; Hoengseong, Republic of Korea). One hundred pupae were placed on filter paper soaked in water in a Petri dish. The experimental methods for (1) cold alone, (2) fumigation alone, and (3) combined treatments were as follows [5,32]. Cold treatment was performed at 1 °C for 24 h. PH_3_ (lethal concentration time; LCT_50_, 1.1 mg/L) was introduced at 20 °C for 4 h in a 12 L desiccator (Bibby Scientific, Staffordshire, UK) sealed with a glass stopper. The pupae were fumigated for 4 h and then immediately exposed to cold air at 1 °C for 24 h. Pupae from each group were transferred to glass vials and rapidly cooled in liquid nitro-gen to prevent metabolic changes. All treatments and controls were triplicated.

### 2.3. Metabolite Extraction

Whole metabolites were extracted from *D. suzukii* pupae in triplicate (100 insects/replicate). Briefly, each sample was suspended in 1 mL of the extracted solution (3:3:2, acetonitrile/isopropyl alcohol/water, *v*/*v*/*v*) and homogenized using a Taco Prep bead beater (Taco, Taichung, Taiwan) while turning it on and off at 30 s intervals for 5 min. Samples were incubated at room temperature for 20 min and centrifuged at 2500× *g* for 5 min at 4 °C. The supernatant was transferred to a new tube and dried under pure N_2_ gas. All dried samples were suspended in 200 μL of 50% acetonitrile and sonicated for 5 min. The supernatant was filtered with 0.22 μm pore (Ultrafree-MC, Millipore, Bedford, MA, USA) and immediately loaded into the LC–QTOF/MS for metabolome analysis. The metabolite recovery rate of the sample was investigated with internal standards (L-alanine, Sigma–Aldrich, Oakville, ON, Canada), and the extraction process showed a recovery rate of 50% or greater.

### 2.4. Lipid Extraction

Total lipidomes were extracted from whole bodies of *D. suzukii* pupae in triplicate (100 insects/replicate) using the modified Bligh and Dyer method, as described previously [33]. Briefly, each sample was suspended in 3 mL of extracted solution (2:1, methanol/chloroform, *v*/*v*) and homogenized using glass beads by turning the beater on and off at 30 s intervals for 5 min. Samples were incubated at room temperature for 20 min and centrifuged at 1750× *g* for 10 min at 4 °C. Supernatants were transferred to new tubes to remove tissue debris. One milliliter of chloroform and 1.8 mL of water were added to each sample, and the mixture was vortexed for 1 min. The lower layer was separated by centrifugation at 1750× *g* for 10 min at 4 °C, followed by transferring to a new tube and drying under pure N_2_ gas. Dried samples were then suspended in 200 μL of loading solution (1:1, methanol/chloroform, *v*/*v*) and sonicated for 5 min. Resulting supernatants were filtered with 0.22 μm pore filters and immediately loaded into the LC–QTOF/MS equipment for lipidomics. Lipid recovery rates for samples were investigated using lipid standards (SPLASH^®^ LIPIDOMIX^®^ Mass Spec Standard, Avanti Polar Lipids, Alabaster, AL, USA), and the extraction process showed recovery rates of 50% or greater [34].

### 2.5. LC-QTOF/MS

LC-QTOF/MS was performed using a liquid chromatograph triple quadrupole mass spectrometer (Agilent Technologies 1260 and 6530 System, Agilent Technologies, Santa Clara, CA, USA; Metabolomics Research Center for Functional Materials, Kyungsung University) with an electrospray ionization (ESI) source. For metabolome analysis, 5 μL of each sample was injected onto a ZORBAX Eclipse XDB-C18 column (4.6 mm × 50 mm, 1.8 μm; Agilent Technologies, Santa Clara, CA, USA) with a temperature of 55 °C. In the binary mobile phase system, phase A was water with 0.1% formic acid and phase B was acetonitrile with 0.1% formic acid. The mobile phase with a flow rate of 0.5 mL/min had the following composition conditions: initiation at 2% B, followed by a linear gradient to 2% B over 1 min, 100% B at 8 min, 100% B at 10 min, 2% B at 11 min, and 2% B at 20 min. Mass spectrometry was performed in both positive and negative modes. The capillary voltage was set to 2.0 kV in the positive mode and 1.0 kV in the negative mode. Metabolites with a mass range of *m/z* 100 to 1000 were detected using a quadrupole time-of-flight instrument.

### 2.6. Data Processing and Statistical Analysis

The data were analyzed in one batch to ensure that the parameters were applied equally to all samples and normalized to the total ion intensity. All entities were extracted from the LC peaks of each sample and analyzed using the Mass Hunter Qualitative soft-ware (Ver. 10.0, Agilent Technologies). All compounds were annotated using the METLIN metabolite database, filtered, scaled, and integrated using Mass Profiler Professional software (Ver. 14.0; Agilent Technologies), principal component analysis (PCA) and Pearson’s correlation analysis were performed. Differentially regulated metabolites were defined as changes in compounds with values of [raw fold change (FC)] > 2 and *p* < 0.01, compared to the mock control group. Metabolites were evaluated using MetaboAnalyst 6.0 (https://www.metaboanalyst.ca) (accessed on 1 August 2024) and LIPEA (https://hyperlipea.org/home) (accessed on 1 August 2024), and relevant pathways were visualized using the Kyoto Encyclopedia of Genes and Genomes (KEGG).

## 3. Results and Discussion

### 3.1. Metabolite Changes according to Stress Conditions

Comparative metabolomics was performed to investigate the physiological effects of low temperature, PH_3_, and combined treatments (low temperature and PH_3_) on *D. suzukii*. When analyzing the total ion chromatogram, peaks that specifically increased or decreased compared to the control were found in each treatment group (Supplementary Figure S1A). By analysis of the mass pattern at 1.57 min, the peak was identified as L-isoleucine, which was only found in the control (Supplementary Figure S1B). A recent study found that transient isoleucine deprivation enhanced nicotine resistance and extended the lifespan of *Drosophila melanogaster* [35]. These results suggest that stresses such as low temperature and PH_3_ exactly affected amino acid synthesis in the *Drosophila* metabolic network. PCA was performed using raw FC data to investigate the reliability of the metabolic analysis (Supplementary Figure S2A). The PCA revealed an aligned cluster of metabolic data for each group and showed a significant distribution pattern in the positive (Supplementary Figure S2A-i) and negative ion modes (Supplementary Figure S2A-ii). Since the correlation analysis showed an association between each experimental group, it can be used to determine treatment-specific indicators based on altered metabolites. Low temperature, PH_3_, and the combined treatment were correlated with each other and revealed the same pattern in the positive (Supplementary Figure S2B-i) and negative ion modes (Supplementary Figure S2B-ii). These results suggest that the metabolome of *D. suzukii* is clearly differentiated by low temperature, PH_3_, and combined treatment.

### 3.2. Pathway Impact of Altered Metabolites

**Figure 1:**
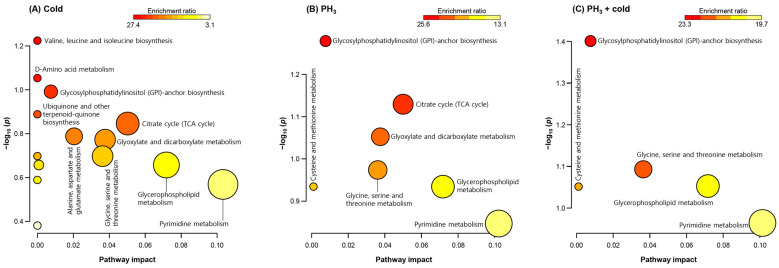
Enrichment ratio and pathway impact scores. Metabolite set enrichment analysis in altered metabolites. (**A**) Low temperature, (**B**) PH_3_, and (**C**) combined treatment. Analysis was performed using the Kyoto Encyclopedia of Genes and Genomes database.

In total, 164 and 98 metabolites were detected in the positive and negative ion modes, respectively, and 80 indicators were filtered using an annotation process based on the metabolite database. Among them, 52 metabolites with significant differences in expression were selected for analysis of their metabolic pathways and were listed as treatment-specific indicators. Metabolites were analyzed using the enrichment ratio and pathway impact scores based on the KEGG database to examine the importance of altered metabolites in the *Drosophila* metabolic network (Figure 1). GPI-anchor and amino acid biosynthetic pathways were significantly regulated under each stress condition. In addition, metabolites related to purine and pyrimidine metabolism were modulated. The tricarboxylic acid (TCA) cycle was found to be the major metabolic network at low temperatures and PH_3_ alone (Figure 1A,B), but could not be identified in the combined treatment (Figure 1C). Interestingly, fewer metabolic pathways were altered in the combined treatment than in the low temperature or PH_3_. The reasons for these results are as follows: (1) only a limited number of metabolites were synergistically affected by the combined treatments; and (2) the combined treatment resulted in significant metabolic changes upon PH_3_ treatment prior to cold exposure.

### 3.3. Treatment-Specific Metabolites as Biomarkers

Since enrichment and pathway impact scores were evaluated for the metabolites found under each stress condition, these results only showed overall tendencies. Therefore, alignment was performed to confirm which metabolites changed quantitatively in response to stress.

Metabolites detected in all treatments, but not in the mock control, were extracted as candidate treatment-specific indicators (Table 1). Metabolites involved in arachidonic acid metabolism and the immune system were identified in the PH_3_ treatment. Interestingly, 3-phosphohydroxypyruvate, an intermediate between 3-phosphoglycerate and pyruvate, was detected in all stresses. 3-phosphohydroxypyruvate generates α-ketoglutarate, a major TCA cycle intermediate, during its conversion to 3-phosphoserine [36]. 3-phosphoserine generates the intermediate serine and the final product glycine. Glycine then binds to the TCA cycle intermediate succinyl-CoA. The interconversion of glutamate to α-ketoglutarate produces various amino acids, including alanine, aspartate, and arginine. Recent studies showed that low temperature and PH_3_ are closely related to energy metabolism [37,38]. PH_3_ induces nerve excitement by acting on acetylcholine, resulting in excessive energy consumption. Therefore, stress-inducing conditions may stimulate the production of intermediate metabolites of pyruvate, and their overproduction has a clear impact on energy metabolic pathways.

In addition, insects respond to stress by inhibiting or inactivating metabolic pathways. Therefore, the up- or downregulation of metabolites compared to the control was sorted because they can be used as indicators for each stress. The amino acids D-proline (Pro) and L-isoleucine (Ile) were detected in quantitative amounts in the mock control but not in the stressed groups. Suppression of the cryoprotectants Pro and Ile, which are known to accumulate in response to the cold in *D. melanogaster*, was contrary to previous results [39,40,41]. Interestingly, the downregulation of aconitic acid, an intermediate product of the TCA cycle, by low temperature and PH_3_, respectively, revealed an improved inhibitory effect.

In invertebrates, PH_3_ increases the signaling of the excitatory neurotransmitter acetylcholine by inhibiting acetylcholine esterase [15]. Persistent synaptic signaling by acetylcholine leads to hyperactivity, convulsions, and ultimately, excitotoxicity. PH_3_ directly interferes with mitochondrial respiration and causes a lack of energy metabolism, which can be confirmed by a decrease in oxygen consumption after 4 h of exposure to PH_3_ [42,43]. In addition, PH_3_ acts as a reducing agent, inhibiting cytochrome c oxidase and inducing the production of hydrogen peroxide, which is a reactive oxygen species (ROS) [44,45]. These PH_3_ responses ultimately resulted in metabolic inhibition, thereby supporting our finding that many metabolites were reduced or suppressed by PH_3_. Collectively, these results suggest that low temperature and PH_3_ share similar metabolic mechanisms that inhibit mitochondrial function and downregulate cellular metabolism.

### 3.4. Comparative Lipidomic Profiling by Stress

**Figure 2:**
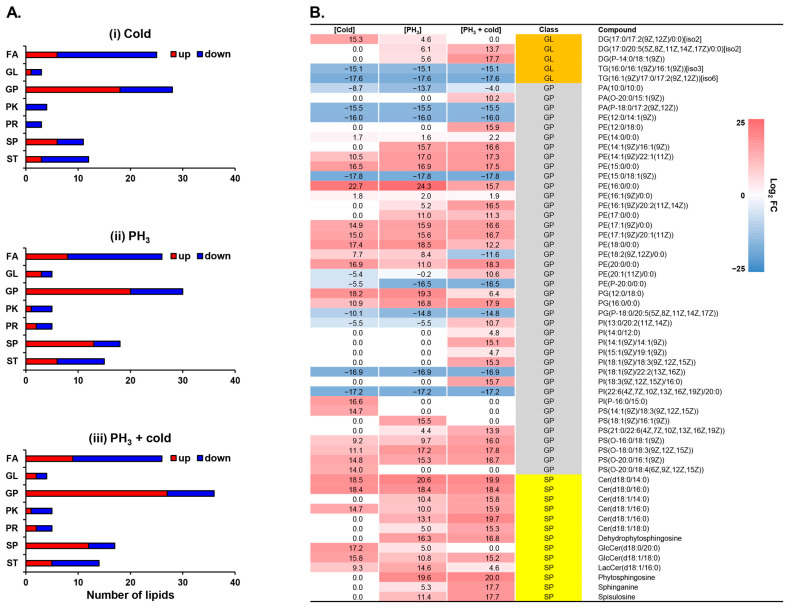
Lipidomic profiling altered by exposure to low temperature and PH_3_. (**A**) The number of lipids showing relative increases and decreases in *D. suzukii* after stress. (**B**) Heatmap of membrane-associated lipids. FA: fatty acid; GL: glycerolipid; GP: glycerophospholipid; PK: polyketide; PR: prenol lipid; SP: sphingolipid; ST: sterol lipid.

Pathway analysis revealed that the metabolites involved in glycosylphosphatidylinositol (GPI)-anchor biosynthesis were significantly regulated by low temperatures, PH_3_, and combined stress (Figure 1). GPI-anchors are covalently linked to the carboxyl terminus of proteins and mediate protein attachment to lipid bilayers [46,47]. GPI, a lipid anchor for cell surface proteins, is associated with lipid rafts enriched in sphingolipids and cholesterol. Therefore, to investigate the changes in lipid profiles in response to stress, 116 lipids were identified through multivariate statistical analysis and annotation (Figure 2). PCA and correlation analyses showed that the clusters of each stress were well aligned and clearly distinguished from the mock control (Supplementary Figure S3). Most lipid classes were quantitatively altered, including fatty acids (FAs), glycerophospholipids (GPs), sphingolipids (SPs), and sterol lipids (STs), but not glycerolipids (GLs), polyketides (PKs), or prenols (PRs) (Figure 2A).

In this study, each stress condition revealed significant regulation of cell surface-related lipids, such as GPs and SPs (Table 1 and Figure 2A). A recent study has shown that lipids provide an energy source for PH_3_-resistant insects to survive and an environment suitable for protecting mitochondria from PH_3_ [48]. In *D. suzukii*, phospholipids in the cell membrane are mainly composed of phosphatidylethanolamine (PE) and a GP class, and low temperatures cause quantitative differences [49]. SPs, components of lipid rafts, are involved in cell membrane receptors and signal transduction, and low temperatures cause changes in the structure and profile of lipid rafts [50,51,52]. Low temperatures can induce changes in the phospholipid bilayer properties of cell membranes, thereby damaging their integrity [53]. These changes in membrane fluidity can lead to neuromuscular dysfunction, chills coma, and ultimately death [54,55,56,57]. Therefore, the altered levels of cell surface-related lipid GLs, GPs, and SPs are presented for each type of stress (Figure 2B). Overall, many metabolites were upregulated compared to the mock control. Heatmap analysis showed that sphingolipids were upregulated by stress and were synergistically affected by the combined treatment. Interestingly, in the GP class, PE and PS were upregulated by the combination treatment, whereas PA, PG, and PI were downregulated. Lipids are the main components of the fat body in insects and most lipids are stored in the form of triglycerides (TGs) [58,59]. In contrast, the major lipid diglyceride (DG) in insect hemolymph increases rapidly during energy requirements such as flight [59,60]. Considering the mechanism of action of PH_3_ in relation to energy depletion, the increase in DG and decrease in TG in response to stress suggests that PH_3_ affects the energy metabolic pathways of *D. suzukii*.

**Figure 3:**
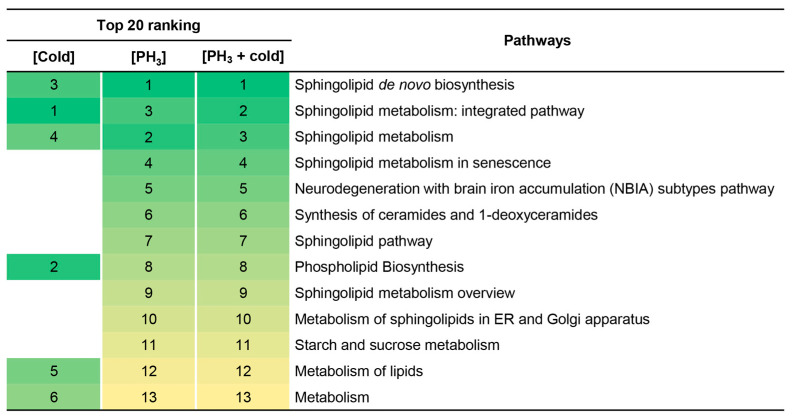
Top 20 signaling pathways enriched at low temperature, PH_3_, and combined treatment. Numbers and colors indicate the ranking (high: green; low: yellow) of the respective signaling pathways.

In addition, the metabolome set enrichment analysis revealed that sphingolipid-related metabolic pathways were primarily affected by stress (Figure 3). There was no difference between the fumigant alone and the combined treatment, but this result may be due to the effect of PH_3_ already prior to the mechanism of action of low temperature on sphingolipids. These results support the reason why fewer metabolic pathways were changed in combined treatment (Figure 2).

## 4. Conclusions

Since studies on fumigants or low temperatures in the *D. suzukii* model are individual, research on metabolic mechanisms is required to understand the synergistic effect of PH_3_, which inhibits cytochrome oxidase activity, induces ROS production, and regulates metabolism at low temperatures. Most metabolites acted on *D. suzukii* metabolic pathways related to amino acid, lipid, and energy biosynthesis. In particular, the synergistic alteration of aconitic acid metabolites involved in the TCA cycle may be an important indicator of physiological changes in *D. suzukii*. In addition, the altered levels of the cell membrane lipids GP and SP revealed the synergistic effect of PH_3_ and low temperatures. Since these metabolites were specifically detected in each stress condition, they can be used as indicators to determine whether the treatment was successfully performed. Therefore, this study provides useful information on treatment-specific biomarkers for low temperature or fumigation.

## Figures and Tables

**Table 1:** Metabolites specifically found in each stress.

KEGG ID (PubChem CID)	Compound	Fold Change (log_2_)				
		[Mock]	[Cold]	[PH_3_]	[PH_3_ + Cold]	Related Pathways
C15675	Myxothiazol Z	-	5.25	-	15.82	Lipids: Polyketides
C05954	19-Hydroxy-PGB_2_	-	-	15.65	17.28	dme00590 Arachidonic acid metabolism
C00350	PE(18:4(6Z,9Z,12Z,15Z)/18:4(6Z,9Z,12Z,15Z))	-	5.09	15.62	15.81	dme00563 Glycosylphosphatidylinositol (GPI)-anchor biosynthesis dme00564 Glycerophospholipid metabolism
C03232	Phosphohydroxypyruvic acid (=3P-hydroxypyruvate)	-	18.40	18.63	18.38	dme00260 Glycine, serine, and threonine metabolism
C01092	8-Amino-7-oxononanoic acid	11.43	-	17.68	17.33	dme00780 Biotin metabolism
52924812	PE(22:4(7Z,10Z,13Z,16Z)/17:1(9Z))	5.98	-	17.91	17.59	Lipids: Glycerophospholipids
C00417	Aconitic acid (=cis-Aconitate)	19.89	19.94	6.43	-	dme00020 Citrate cycle (TCA cycle)
(614)	D-Proline	23.07	15.37	-	-	map00470 D-Amino acid metabolism
C00407	L-Isoleucine	24.81	7.50	-	-	dme00280 Isoleucine degradation dme00290 Isoleucine biosynthesis
C04778	N1-(5-Phospho-a-D-ribosyl)-5,6-dimethylbenzimidazole	15.93	10.50	-	-	dme00860 Porphyrin metabolism
C03794	N6-(1,2-dicarboxyethyl)-AMP	9.85	15.31	-	-	dme00230 Purine metabolism
(52924712)	PE(21:0/20:5(5Z,8Z,11Z,14Z,17Z))	16.66	10.06	-	-	Lipids: Glycerophospholipids
C00156	4-Hydroxybenzoic acid (=*p*-Salicylic acid)	17.06	11.37	-	-	dme00130 Ubiquinone biosynthesis
(135398700)	Xanthopterin	16.22	10.74	-	-	dme00790 Folate biosynthesis
(5312441)	13Z-Octadecenoic acid	4.61	-	-	-	dme00061 Fatty acid biosynthesis
(53480926)	LysoPE(0:0/18:2(9Z,12Z))	21.28	-	-	-	Lipids: Glycerophospholipids
(42607464)	PE(17:1(9Z)/0:0)	18.14	-	-	-	Lipids: Glycerophospholipids
C00366	Uric acid	22.22	-	-	-	dme00230 Purine metabolism

## Data Availability

The data presented in this study are available in insert article or supplementary material here.

## Supplementary Materials

The following supporting information can be downloaded at: https://www.mdpi.com/article/10.3390/metabo14100526/s1, Figure S1: Total ion current profile and distribution of altered metabolites; Figure S2: Comparative analysis of expression patterns between stresses according to metabolic changes; Figure S3: Comparative analysis of expression patterns between stresses according to lipidomic changes.
