# Supplementary material for: Comparative Metabolic Profiling in Drosophila suzukii by Combined Treatment of Fumigant Phosphine and Low Temperature
Source: Metabolites. 2024 Sep 28;14(10):526. doi: 10.3390/metabo14100526 (PMC11509839; doi:10.3390/metabo14100526)

Comparative metabolic profiling in *Drosophila suzukii* by combined treatment of fumigant phosphine and low temperature

Junbeom Lee, Hyun-Kyung Kim, Jong-Chan Jeon, Seung-Ju Seok, Gil-Hah Kim, Hyun-Na Koo and Dae-Weon Lee

SUPPLEMENTARY DATA

Supplementary Figure S1. Total ion current profile and distribution of altered metabolites.

(A) LC and (B) LC-MS spectral analysis under stressful conditions. Purple arrows indicate the same retention time.

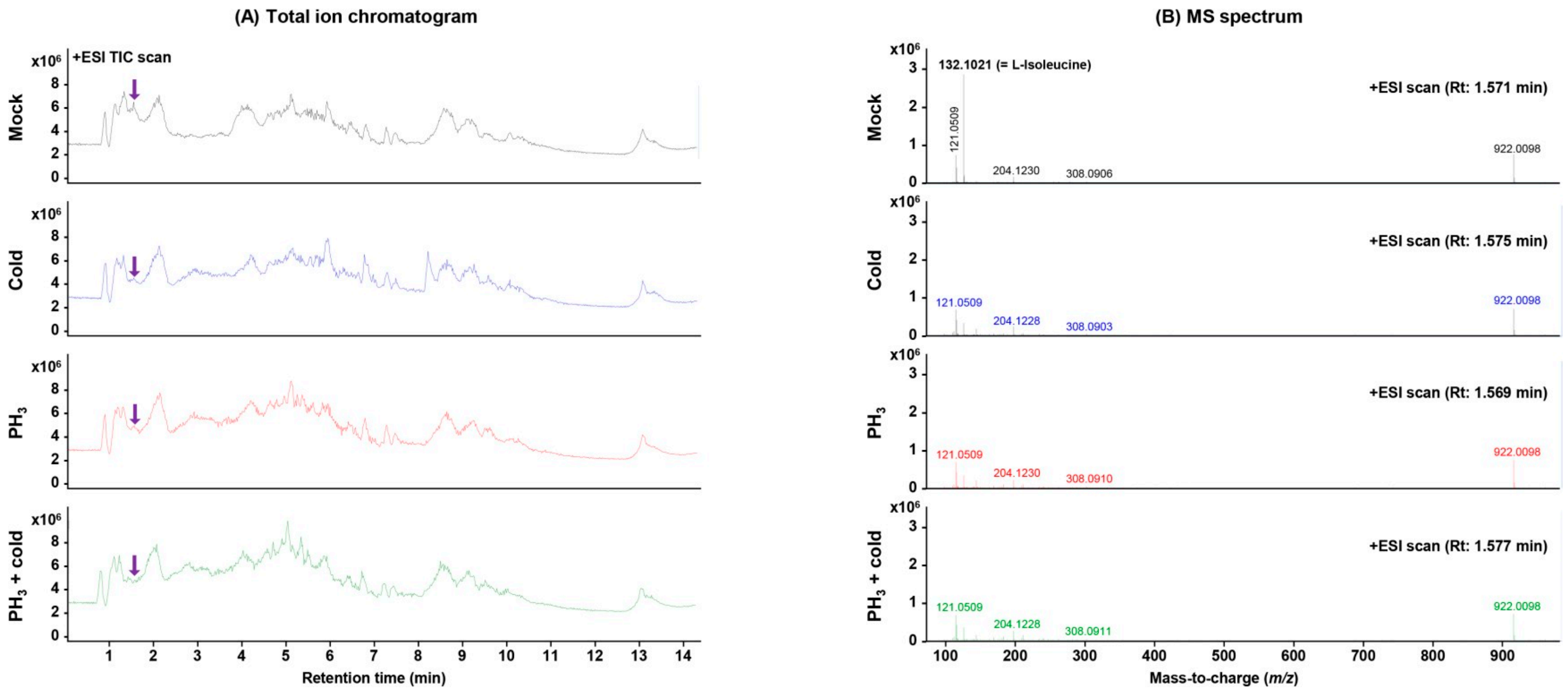

Supplementary Figure S2. Comparative analysis of expression patterns between stresses according to metabolic changes.

(A) PCA and (B) correlation plots between experimental groups with altered metabolites in (i) positive and (ii) negative ion mode. Each colored dot indicates the number of repetitions (n = 3).

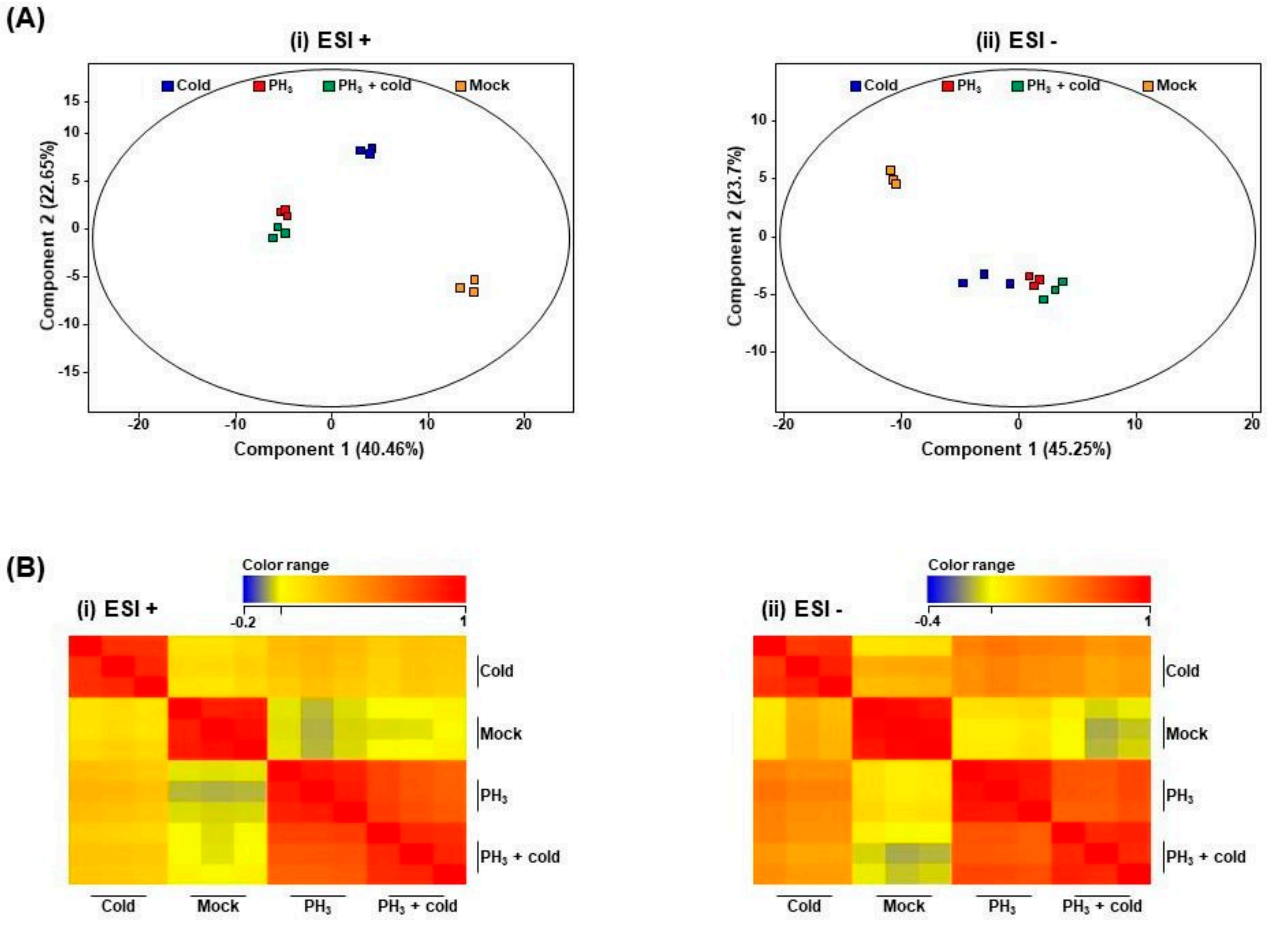

Supplementary Figure S3. Comparative analysis of expression patterns between stresses according to lipidomic changes.

(A) PCA and (B) correlation plots between experimental groups with altered lipids in (i) positive and (ii) negative ion mode. Each colored dot indicates the number of repetitions (n = 3).

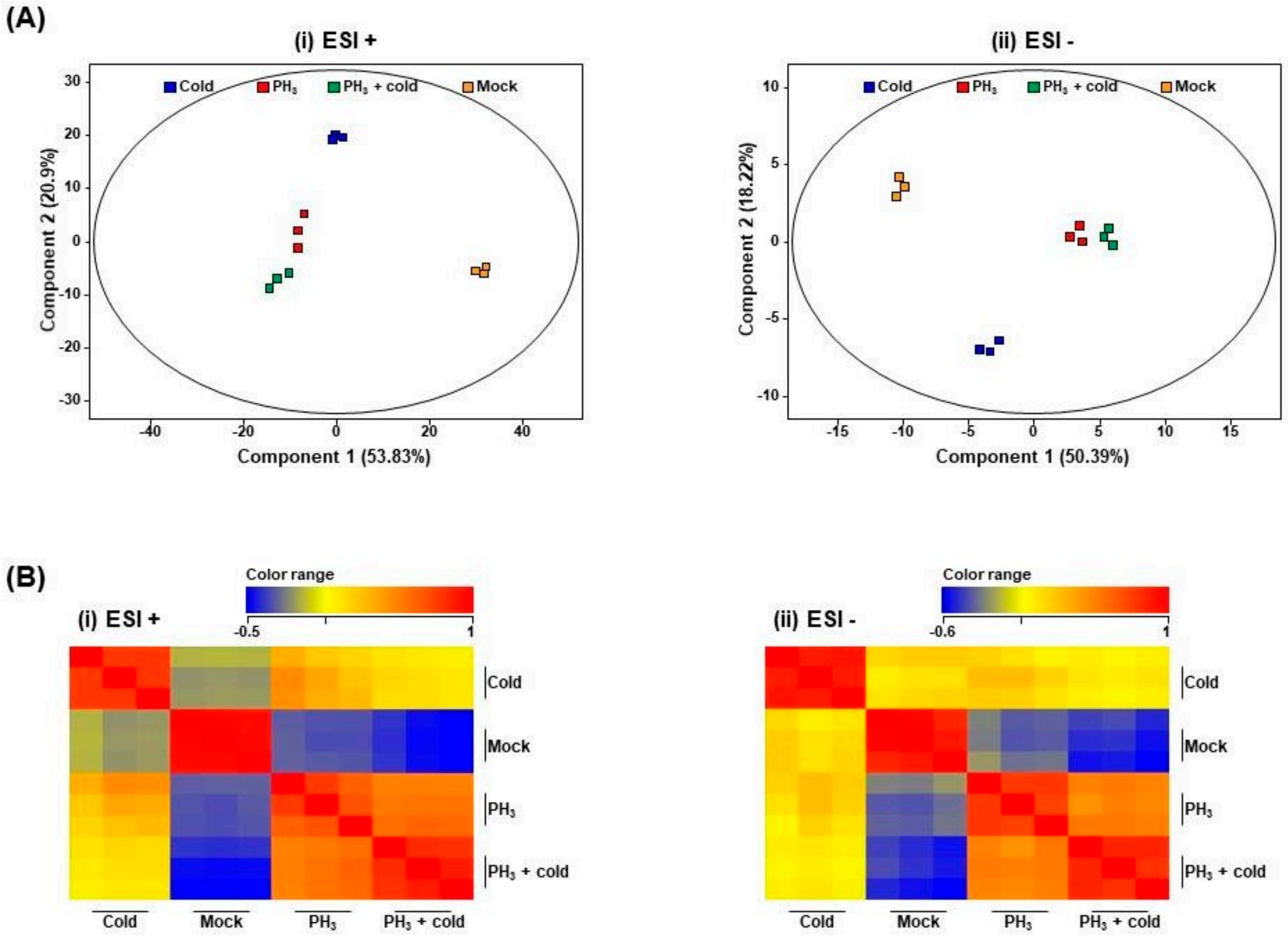

Supplement: Supplementary file 1 [file metabolites-14-00526-s001.zip › metabolites-3217130-supplementary.pdf]
